# New aspects of antiproliferative activity of 4-hydroxybenzyl isothiocyanate, a natural H_2_S-donor

**DOI:** 10.1007/s00726-018-2546-2

**Published:** 2018-03-05

**Authors:** Halina Jurkowska, Maria Wróbel, Dominika Szlęzak, Ewa Jasek-Gajda

**Affiliations:** 1 Chair of Medical Biochemistry Jagiellonian University Medical College, 7 Kopernika St., 31-034 Kraków, Poland; 2 Department of Histology, Jagiellonian University Medical College, 7 Kopernika St., 31-034 Kraków, Poland

**Keywords:** 4-Hydroxybenzyl isothiocyanate, Hydrogen sulfide, Thiosulfate, Apoptosis, Sulfurtransferases, Cancer cells

## Abstract

The effect of 4-hydroxybenzyl isothiocyanate (HBITC), a natural H_2_S-donor from white mustard seeds (*Sinapis alba*), on the proliferation of human neuroblastoma (SH-SY5Y) and glioblastoma (U87MG) cells was studied and some aspects of the mechanism of its activity were suggested. The inhibition of both SH-SY5Y and U87MG cell proliferation was associated with an increase in the thiosulfate level, the number of cells with the inactive form of Bcl-2 protein, and with a decrease of mitochondrial membrane potential. Interestingly, HBITC results in downregulation of p53 protein and upregulation of p21 protein levels in SH-SY5Y cells. In the presence of elevated levels of H_2_S and thiosulfate, the sulfhydryl groups of p53 protein as well as Bcl-2 protein could be modified via HBITC-induced S-sulfuration or by oxidative stress. It seems that the induction of p21 protein level is mediated in SH-SY5Y cells by p53-independent mechanisms. In addition, HBITC-treatment caused downregulation of the level of mitochondrial rhodanese and 3-mercaptopyruvate sulfurtransferase, and consequently increased the level of the reactive oxygen species in SH-SY5Y cells.

## Introduction

Hydrogen sulfide (H_2_S) is endogenously generated in mammalian cells via enzymatic and non-enzymatic pathways. The enzymatic pathways generate H_2_S from l-cysteine and L-homocysteine using cystathionine-β-synthase (CBS), cystathionine-γ-lyase (CTH), and cysteine aminotransferase with 3-mercaptopyruvate sulfurtransferase (MPST). H_2_S can be also produced from d-cysteine by MPST with an earlier conversion by D-amino acid oxidase. Non-enzymatic production of H_2_S can occur through glutathione-dependent conversations (Szabo and Papapetropoulos 2017; Tkacheva et al. 2017; Rose et al. 2017; Yagdi et al. 2016; Zheng et al. 2015; Zhang et al. 2017a). H_2_S is oxidized by sulfide quinone oxidoreductase (SQR) into a sulfane sulfur atom, which is next transferred to sulfite forming thiosulfate. Sulfane sulfur atoms from thiosulfate can be transferred to reduced glutathione (GSH) by rhodanese (thiosulfate sulfurtransferase, TST) generating glutathione persulfide (GS-SH). Mitochondrial sulfur dioxygenase converts the sulfane sulfur persulfide forming sulfite. Then, sulfite is oxidized to sulfate by sulfite oxidase or is degraded by SQR to produce thiosulfate (Yagdi et al. 2016; Kabil et al. 2014; Jurkowska et al. 2014).

In the human body, natural hydrogen sulfide donors, releasing H_2_S non-enzymatically or enzymatically, include organic polysulfides from garlic (*Allium sativum*) or isothiocyanates from brassicas (Brassicaceae) (Tkacheva et al. 2017; Szabo and Papapetropoulos 2017). These natural products have shown interesting properties, such as anti-microbial, anti-thrombotic, anti-oxidant and anti-tumor (Song et al. 2014; Gupta et al. 2014a; Dufour et al. 2015). However, some of these effects of H_2_S, i.e. the anti-cancer potential, remain controversial and some of them depend on its concentration (Baskar and Bian 2011; Hellmich et al. 2015; Yagdi et al. 2016).

Citi et al. (2014) for the first time reported that some naturally occurring isothiocyanates (allyl isothiocyanate, 4-hydroxybenzyl isothiocyanate, benzyl isothiocyanate, and erucin) behave as a slow H_2_S-releasing agent. Among these compounds, 4-hydroxybenzyl isothiocyanate (HBITC), a natural product obtained from white mustard seeds *(Sinapis alba)*, was the most effective, exhibiting significant and remarkable H_2_S-release, both in the absence and in the presence of l-cysteine (Citi et al. 2014).

Based on these data, we decided to choose HBITC to investigate the effects and mechanisms of its action in the regulation of human neuroblastoma (SH-SY5Y) and glioblastoma (U87MG) cell proliferation. It was previously demonstrated by Jurkowska et al. (2017) that diallyl trisulfide, a garlic-compound, could inhibit proliferation of these cells by its effect on Bcl-2 protein. The inhibition of U87MG cell proliferation was correlated with an increased level of sulfane sulfur (in these cells, the level of l-cysteine is higher than in SH-SY5Y cells) (Jurkowska et al. 2017).

In the present study, it was shown for the first time that HBITC, a natural H_2_S-donor, could reduce proliferation of both SH-SY5Y and U87MG cells. This effect was correlated with the increased level of thiosulfate, so it seems that thiosulfate as a product of H_2_S oxidation, and on the other hand a donor of sulfane sulfur, can be involved in the mechanisms that lead to inhibition of cell proliferation. The antiproliferative activity of HBITC was also associated with downregulation of mitochondrial rhodanese and MPST protein levels, a loss of mitochondrial membrane potential, inactivation of Bcl-2 protein and modulation (S-sulfuration, oxidative stress) of proteins involved in the cell cycle.

## Materials and methods

### Sources of chemicals

Crystal violet (*N*-hexamethylpararosaniline), hexadecyltrimethylammonium bromide (CTAB) and albumin were obtained from Sigma-Aldrich Corp. (St. Louis, MO, USA). Trypsin, and penicillin/streptomycin were obtained from HyClone Laboratories (Utah, USA). The Cytotoxicity Detection Kit (LDH) and the Cell Proliferation ELISA, BrdU (colorimetric) Kit were obtained from Roche Applied Science (Germany). Potassium cyanide (KCN) was obtained from Merck (Darmstadt, Germany). All the other chemicals were of reagent grade and purchased from common commercial suppliers.

### Cell culture

Human SH-SY5Y and U87MG cells (ECACC, UK) were grown in Dulbecco’s Modified Eagle’s (DMEM) medium (Biosera, France) containing stable l-glutamine, 4500 mg/l glucose and sodium pyruvate, to which 10% fetal bovine serum (FBS) (Biowest, South America) and 1% penicillin/streptomycin (100 Units/ml penicillin and 100 µg/ml streptomycin) were added. The cells were maintained at 37 °C in a humidified atmosphere containing 5% CO_2_.

4-Hydroxybenzyl isothiocyanate (Santa Cruz Biotechnology, Texas, USA) was dissolved in dimethyl sulphoxide (DMSO; Sigma-Aldrich Corp., St. Louis, MO, USA), and then diluted with DMEM to the desired concentration prior to its use. The final concentration of DMSO in the medium was less than 0.1%.

### Determination of HBITC cytotoxicity

Cell cytotoxicity was investigated by measuring the leakage of lactate dehydrogenase (LDH) from dead or dying cells using the Cytotoxicity Detection Kit (Roche Applied Science, Germany) according to the manufacturer’s protocol as described previously (Jurkowska et al. 2011). The concentration of HBITC (20 µM, 40 µM, 60 µM, 80 µM) that yielded LDH leakage of less than 5% was used in the experiments.

### Cell proliferation

For the determination of cellular proliferation, the cells were seeded on 96-well plates at a concentration of 1.5 × 10^3^ cells/well (SH-SY5Y cells) and 1.2 × 10^3^ cells/well (U87MG cells) in DMEM supplemented as reported above. Following 24 h of incubation, the culture medium was replaced with 100 µl of complete medium with DMSO (as the controls) or 100 µl of the medium containing various HBITC concentrations (20, 40, 60, 80 µM), and then the cells were cultured for 24 and 48 h. Cell proliferation was examined using the Cell Proliferation ELISA, BrdU (colorimetric) test (Roche Applied Science, Germany) according to the manufacturer’s protocol and also the modified crystal violet staining method (Gillies et al. 1986) was used. The absorbance was measured using an Epoch Microplate Spectrophotometer (BioTek Instruments Inc, VT, USA).

### Detection of H_2_S in cells

A H_2_S fluorescent probe WSP-5 (Cayman Chemical, Michigan, USA) was used for detection of H_2_S released from HBITC, according to the method of Peng et al. (2014). SH-SY5Y and U87MG cells were cultured with HBITC (40, 60, 80 μM) or the same volume of DMSO in the medium (as the controls). Then, the cells were incubated with a H_2_S fluorescence probe WSP-5 (50 μM) and a surfactant hexadecyltrimethylammonium bromide (CTAB, 100 μM) in PBS at 37 °C for 20 min in the dark. After the PBS was removed, H_2_S-derived fluorescence was observed under a fluorescent microscope (Axioplan-2; Carl Zeiss Imaging, Oberkochen, Germany).

### Measurement of thiosulfate

For the measurement of thiosulfate, the samples of the culture medium were deproteinated using Amicon Ultra 0.5-mL centrifugal filters with a 3 kDa cutoff (Millipore, Merck, Poland), centrifuged at 14 000 x g for 20 min and assayed for thiosulfate by the method of Shih et al. (1979).

### Western blot

The cells were plated at a density of 2.5 × 10^4^ per well in six-well plates. On the following day, the cells were treated with various concentrations of HBITC or an equal volume of DMSO in the culture medium for 24 and 48 h. Proteins from the cells were extracted in lysis buffer containing 50 mM Tris–HCl, pH 7.5, 150 mM NaCl, 1 mM EDTA, 0.5% NP-40, and 1X Complete Protease Inhibitor Cocktail (Sigma-Aldrich Corp., St. Louis, MO, USA). Cell lysates were centrifuged at 20 000 x g for 15 min at 4 °C. Protein concentration was determined by the bicinchoninic acid (BCA) method using a BCA Protein Assay Kit (Thermo Scientific/Pierce Biotechnology, Rockford, IL, USA) with bovine serum albumin (BSA) as a standard. The samples (25 µg) were subjected to SDS-PAGE (12%, w/v, polyacrylamide) and the separated proteins were transferred onto polyvinylidene difluoride (PVDF, Bio-Rad, CA, USA) membrane followed by blocking of the membrane with 5% nonfat milk. The sources and dilutions of primary antibodies were as follows: anti-TST (1:800; Proteintech Group, USA), anti-MPST (1:800; GeneTex, Inc., CA, USA), anti-CTH (1:1000; Abnova, Taiwan), anti-p53 (1:800; Upstate & Chemicon, CA, USA), anti-p21 (1:800; Upstate & Chemicon, CA, USA), anti-β-actin (1:1000; Sigma-Aldrich Corp., St. Louis, MO, USA). The membranes were incubated with anti-mouse or anti-rabbit goat antibodies conjugated with alkaline phosphatase (1:2000, Proteintech Group, USA) and the immune complexes were visualized using the NBT/BCIP Stock Solution (Roche Applied Science, Germany). The densities of the bands were quantified using the ChemiDoc^TM^MP Imaging System (Bio-Rad, USA). β-actin was used as the internal control.

### Bcl-2 Expression assay

Bcl-2 expression was analyzed using the Muse^TM^ Bcl-2 Activation Dual Detection Kit (Millipore, Billerica, MA, USA). The assay utilizes two directly conjugated antibodies, a phospho-specific anti-phospho-Bcl-2 (Ser70)-Alexa Fluor 555 and an anti-Bcl-2-PECy5 conjugated antibody to measure total levels of Bcl-2 expression. The cells were prepared for this analysis as described previously (Jurkowska et al. 2017) and were analyzed by a Muse^TM^ Cell Analyzer and a Muse^TM^ analysis software (Merck Millipore, USA).

### Oxidative stress assay

The quantitative measurement of cellular populations undergoing oxidative stress was performed using the Muse Oxidative Stress Kit (Merck Millipore, Billerica, MA, USA) according to the manufacturer’s instructions. This assay utilizes dihydroethidium (DHE), which is cell membrane-permeable and upon reaction with superoxide anions undergoes oxidation to form DNA-binding fluorophore. The kit determines the percentage of cells that are negative [ROS(−)] and positive [ROS(+)] for reactive oxygen species. Briefly, 1 × 10^6^ cells/ml were harvested, washed with PBS, and then incubated in the dark at 37 °C for 30 min with the Muse Oxidative Stress Reagent working solution, which contained DHE. The count and percentage of cells undergoing oxidative stress were quantified using the Muse Cell Analyzer and Muse analysis software (Merck Millipore, USA).

### Mitochondrial transmembrane potential detection assay

Mitochondrial transmembrane potential (MMP, ΔΨm) was determined using the MitoPT™ JC-1 assay kit (Immunochemistry Technologies, LCC, MN, USA) according to the manufacturer’s instructions. JC-1 exists as a green fluorescent monomer at low MMP, while at higher MMP, JC-1 forms red fluorescent aggregates and can therefore be used as a sensitive measure of changes in MMP. Briefly, the cells were resuspended in the MitoPT™ JC-1 solution and incubated in the dark for 15 min at 37 °C. After incubation, the cells were resuspended in 1× assay buffer and seeded in a 96-well clear bottom black plate at a density of 1x10^5^ cells/100 μl per well. Fluorescence was assessed in the microplate reader by measuring the JC-1 aggregates (590 nm emission) following 488 nm excitation (JC-1 monomers). The changes in mitochondrial membrane potential were calculated as the ratio of JC-1 aggregate to monomer fluorescence intensity. The mitochondrial membrane potential in the treated cells was expressed as the percentage of that in the control cells (100%).

### Statistical analysis

The data were expressed as the means ± standard deviation (SD). Statistical analyses were performed using the Student’s *t* test. All the experiments were repeated at least three times. Results with *p* < 0.05 were considered significant.

## Results

### Effects of HBITC on the cell proliferation

**Fig. 1:**
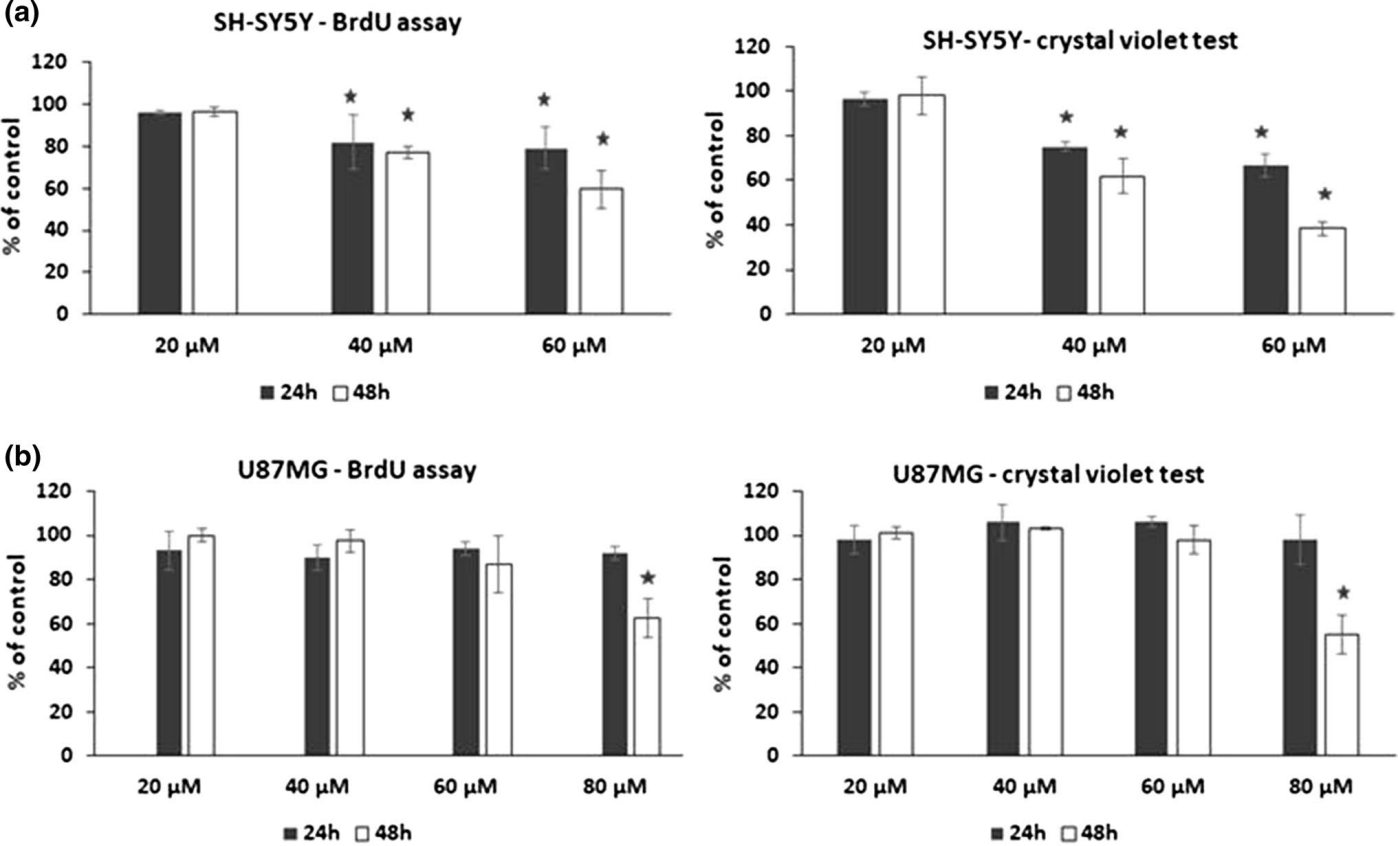
The effect of HBITC on the proliferation of SH-SY5Y (**a**) and U87MG (**b**) cells. SH-SY5Y and U87MG cells were incubated for 24 and 48 h at various concentrations (20, 40, 60, 80 µM) of HBITC. Cell proliferation was analyzed by the BrdU and crystal violet assay. The data presented in the figure are the mean ± SD of four-five independent experiments. *p < 0.05 was considered statistically significant as compared with non-treated controls

To determine the potential antiproliferative effect of HBITC, the human SH-SY5Y and U87MG cells were treated with HBITC for 24 and 48 h, and then the BrdU and crystal violet assays were performed. As compared to the cells cultured without HBITC, the cell proliferation of the treated SH-SY5Y cells was inhibited in a dose- and time-dependent manner (Fig. 1a). The inhibition of SH-SY5Y cell proliferation was significant in case of treatment with 40 μM and 60 μM HBITC for 24 h (~ 20% of inhibition) and 48 h (~ 20 and 40% of inhibition, respectively). U87MG cells were less reactive to HBITC—the concentrations up to 60 µM did not affect the proliferation of these cells. At the higher concentration (80 µM), HBITC inhibited U87MG proliferation by ~ 40% after 48 h of culture (Fig. 1b).

### Effect of HBITC on the H_2_S level

**Fig. 2:**
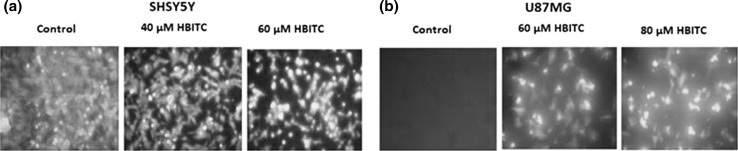
HBITC effect on the H_2_S levels in SH-SY5Y (**a**) and U87MG (**b**) cells. H_2_S fluorescent probe WSP-5 was used to detect H_2_S in the cells. Intracellular H_2_S-derived florescence was observed under a fluorescent microscope (Axioplan-2; Carl Zeiss Imaging, Oberkochen, Germany) connected to a Canon PC1200 camera and AxioVision software (AxioVs40, V 4.6.3.0), and the images were produced

Cell imaging experiments were performed to detect H_2_S in the cells. The cells were incubated with 40 µM and 60 µM HBITC (SH-SY5Y) and with 60 and 80 µM HBITC (U87MG). After incubation with WSP-5 fluorescence probe, strong fluorescence signals were observed (Fig. 2), what confirmed that H_2_S was released from HBITC.

### Effect of HBITC on the thiosulfate level

**Fig. 3:**
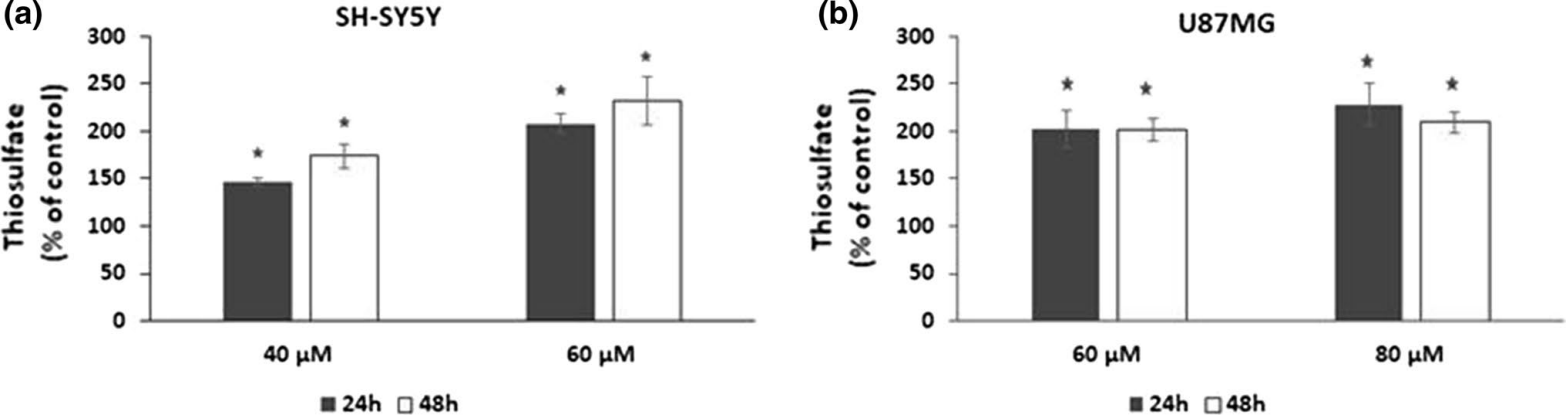
HBITC effect on the thiosulfate levels in SH-SY5Y (**a**) and U87MG (**b**) cells. The cells were incubated for 24 h and 48 h in the presence of various concentrations of HBITC. Every value represents the mean ± SD of four independent experiments; *p < 0.05 (Student’s *t* test). For SH-SY5Y cells, the thiosulfate level determined after 24 and 48 h of culture equaled, respectively, to 103 ± 27 and 109 ± 27 nmole/ml (control values). For U87MG cells, the thiosulfate level determined after 24 and 48 h of culture equaled, respectively, to 117 ± 18 and 153 ± 8 nmole/ml (control values)

The results showed that after treating the cells with HBITC for 24 and 48 h, the thiosulfate level in the culture medium was significantly increased (Fig. 3). The thiosulfate level in SH-SY5Y and U87MG cells was about 1.5–2.5-fold and 2-fold of that of the control group, respectively.

### Effect of HBITC on the level of proteins involved in cell cycle regulation and H_2_S metabolism

**Fig. 4:**
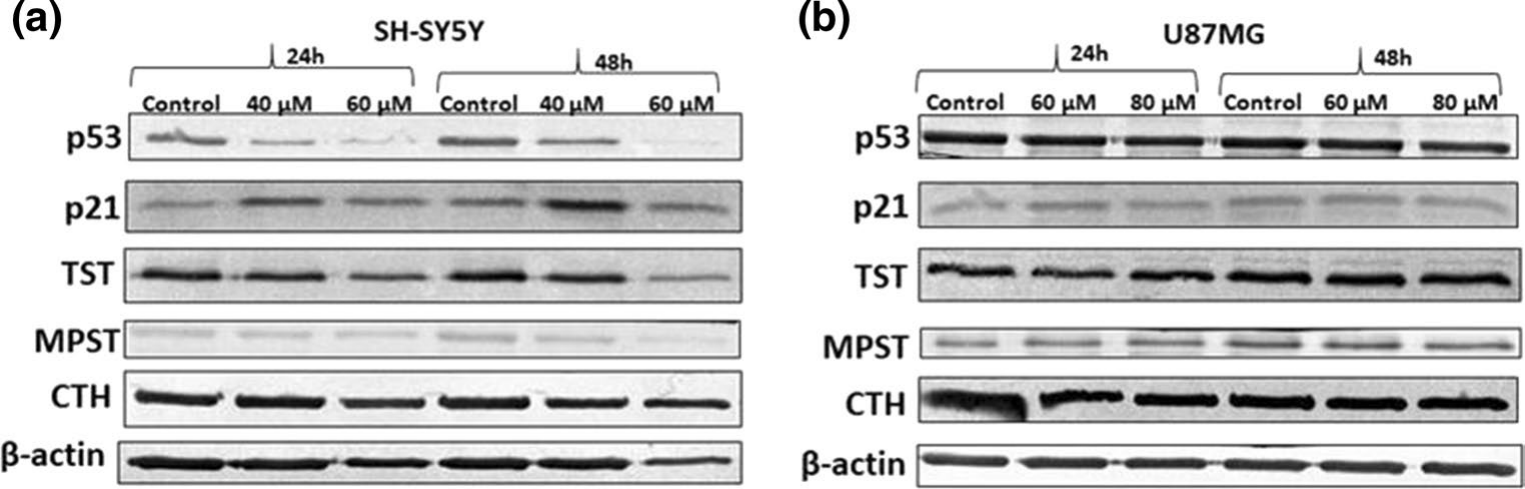
The level of p53, p21, TST, MPST, and CTH proteins in SH-SY5Y (**a**) and U87MG (**b**) cells treated with HBITC. The cells were incubated with HBITC (40 µM, 60 µM, 80 µM) for 24 and 48 h. Protein extraction and Western blot analysis were then performed to determine the level of these proteins. β-actin was used as the loading control. Data from a representative experiment are presented. All the experiments were repeated at least three times with similar results

The Western blot analysis showed that the level of p53, a regulator molecule of cell cycle, was significantly decreased in SH-SY5Y cells upon treatment with HBITC in a dose-dependent manner (Fig. 4a). In addition, the level of p21 was increased after 40 µM HBITC treatment of these cells. Furthermore, the levels of TST and MPST, proteins involved in H_2_S metabolism, were reduced in SH-SY5Y cells treated with HBITC, but the level of CTH sulfurtransferase was unchanged (Fig. 4a). Interestingly, HBITC did not affect the level of these proteins in U87MG cells (Fig. 4b).

### Effect of HBITC on the Bcl-2 expression

**Fig. 5:**
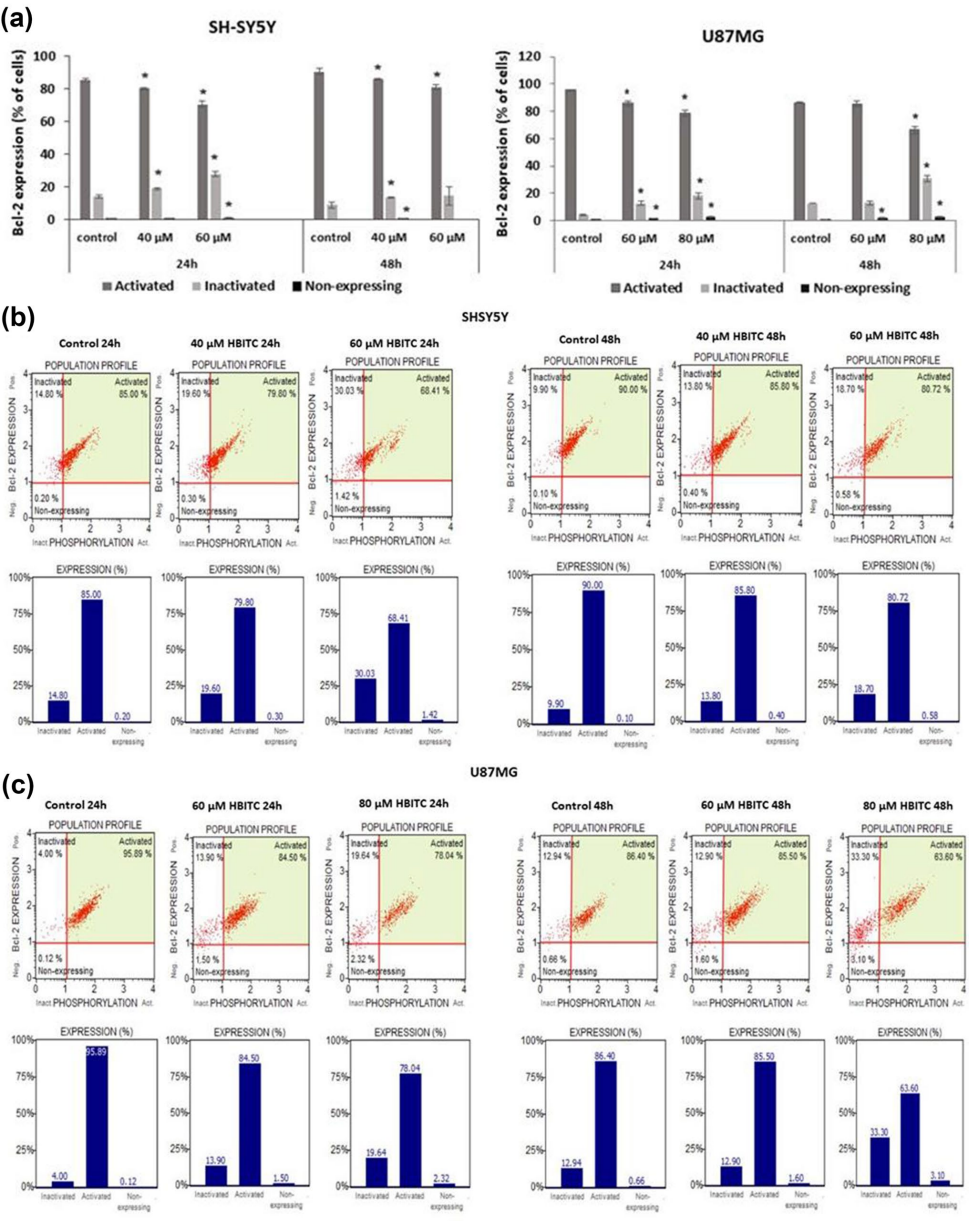
Effect of HBITC on the Bcl-2 expression in SH-SY5Y and U87MG cells. The cells were incubated with 20 µM, 40 µM, 60 µM and 80 µM of HBITC for 24 and 48 h. The percentage of the cells with active and inactive form of Bcl-2, and non-expressing cells was determined by flow cytometry analysis using a Muse^TM^ Bcl-2 Activation Dual Detection Kit. **a** Bar graphs represent the mean ± SD from three-four independent experiments. *p < 0.05 (Student’s *t* test). **b**, **c** Representative results are shown

A Muse^TM^ Bcl-2 Activation Dual Detection Kit was used to measure the percentage of Bcl-2 protein activation in the cells. The percentage of the cells with active and inactive (phosphorylated) form of Bcl-2, and non-expressing cells was determined. As shown in Fig. 5a–c, the active form of Bcl-2 was decreased, but the inactive form of Bcl-2 was increased in both HBITC-treated cancer cell lines (*p < 0.05).

### Effect of HBITC on ROS production

**Fig. 6:**
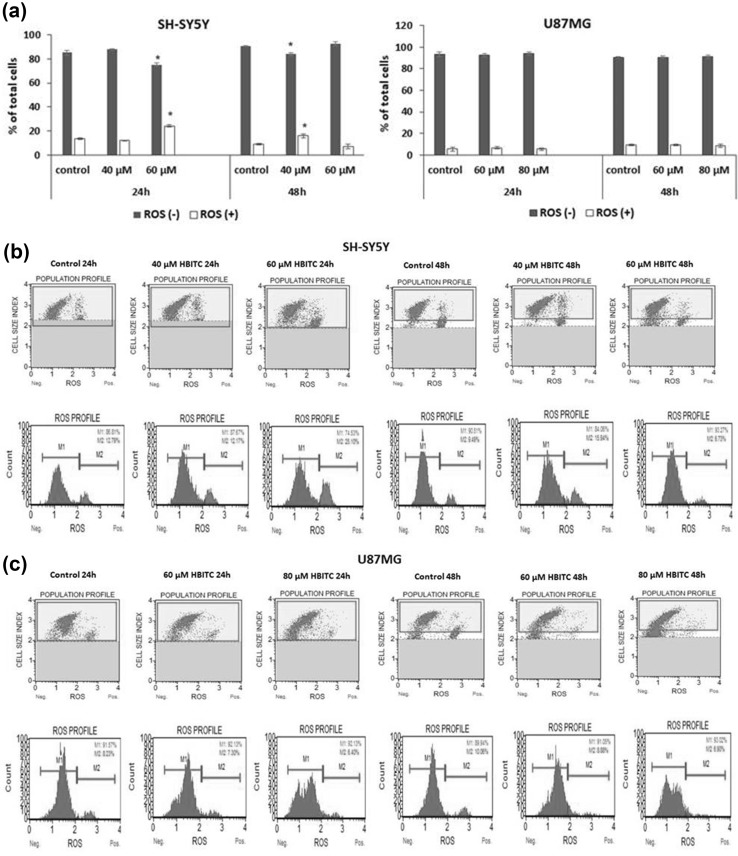
Reactive oxygen species production detected in HBITC-treated SH-SY5Y and U87MG cells. The control cells and the cells treated with HBITC at concentrations of 20, 40, 60, and 80 µM were incubated for 24 and 48 h. The percentage of ROS-positive and ROS-negative cells was determined. **a** Bar graphs represent the mean ± SD from three independent experiments. **b**, **c** Representative results are shown. The blue area (M1) represents the negative cells ROS(−), and the red area (M2) represents the cells with ROS activity [ROS(+)]

The count and percentage of the cells undergoing oxidative stress were measured by flow cytometry using the Muse Oxidative Stress Kit. We observed that HBITC caused an increase in the number of ROS-positive SH-SY5Y cells (Fig. 6a, b). HBITC did not result in ROS elevation in U87MG cells under the experimental conditions (Fig. 6a, c).

### Effect of HBITC on the mitochondrial transmembrane potential (Ψm)

**Fig. 7:**
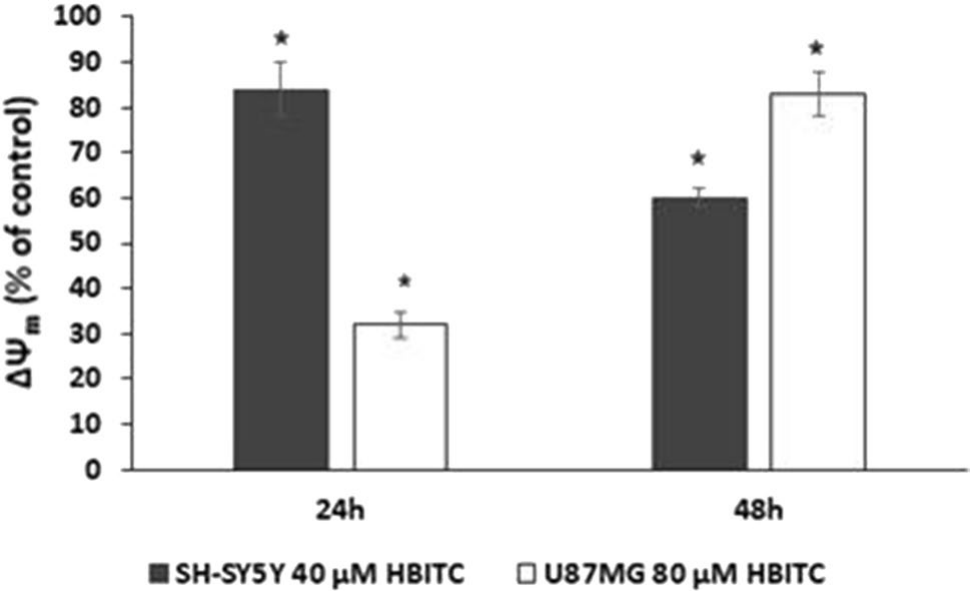
Effect of HBITC on the mitochondrial membrane potential (Ψm) of SH-SY5Y and U87MG cells. The cells were treated with HBITC (SH-SY5Y- 40 µM; U87MG- 80 µM) for 24 and 48 h. The cells were then stained with JC-1 dye and analyzed using a fluorescent microplate reader. Values represent the mean ± SD from three independent experiments. *p < 0.05 (Student’s *t* test)

Apoptosis is often accompanied by mitochondrial dysfunction. To investigate the effect of HBITC on the mitochondrial function of SH-SY5Y and U87MG cells, the indicators of mitochondrial activity, such as ΔΨm, were monitored using JC-1 staining. We observed that the incubation of the cells in the presence of HBITC caused a significant decrease in Ψm of both cancer cells (Fig. 7).

## Discussion

Natural isothiocyanates, such as sulphoraphene, benzyl isothiocyanate (BEITC), phenethyl isothiocyanate (PEITC), and iberin, have anti-cancer properties on prostate (Pawlik et al. 2017), breast (Lin et al. 2017), colon (Lai et al. 2010), glioma (Zhu et al. 2013; Su et al. 2015), neuroblastoma (Jadhav et al. 2007), leukemia and lung (Gupta et al. 2014b) cancer cells.

In the present study, it has been demonstrated for the first time that 4-hydroxybenzyl isothiocyanate results in the inhibition of proliferation of human neuroblastoma SH-SY5Y (Fig. 1a) and glioblastoma U87MG (Fig. 1b) cells. Interestingly, we have shown that the antiproliferative effect of HBITC is associated with an increase in H_2_S (Fig. 2) and thiosulfate levels (Fig. 3). Hydrogen sulfide exists in the equilibrium with HS^−^ (in the ratio 2:8) at physiological pH. Furthermore, H_2_S (and HS^−^) has a very short half-life in biological fluids including cell culture medium and blood (Marutani et al. 2015). Thus, it seems that thiosulfate produced from HBITC, and other products of H_2_S metabolism, can be involved in the mechanisms that lead to inhibition of cell proliferation.

We have found that HBITC has a more potent inhibitory effect on SH-SY5Y cells proliferation compared to U87MG cells, at the same concentrations in these cells (Fig. 1). Lee et al. (2014) reported that H_2_S enhanced glucose uptake and increased glycolytic rate, lactate production, intracellular acidification and cancer cell death (Lee et al. 2014). It appears that U87MG cells, which have a higher than SH-SY5Y cells mitochondrial metabolic capacity, expression of PPAR-γ (peroxisome proliferator-activated receptor-γ) and lactate dehydrogenase (Kim et al. 2015), are to a lesser degree responsible for HBITC-induced changes.

In the present study, the increased H_2_S and thiosulfate levels in HBITC-treated SH-SY5Y cells have been associated with downregulation of the level of rhodanese and MPST, but the level of γ-cystathionase has not been changed in both cancer cell lines (Fig. 4). MPST and rhodanese are localized in the mitochondria and exhibit antioxidant properties (Nagahara 2011; Nagahara et al. 2015). Their catalytic site cysteines are reversibly oxidized by ROS. In the presence of a high level of H_2_S and thiosulfate (Figs. 2 and 3), the sulfhydryl groups of proteins can be modified by S-sulfuration. This process is a post-translational modification of l-cysteine residues (-SH to –SSH), by which H_2_S exerts various biological effects (Zhang et al. 2017b; Toohey 2012; Mustafa et al. 2009). H_2_S induces S-sulfuration of enzymes or receptors, transcription factors, and ion channels (Meng et al. 2017). Marutani et al. (2015) suggested that S-sulfuration was involved in inhibition of caspase-3 in SH-SY5Y cells caused by sodium sulfide and thiosulfate. Downregulation of mitochondrial MPST and TST protein levels observed in our study results in the increased ROS level in SH-SY5Y cells after treatment with HBITC (Fig. 6). Our previous studies (Jurkowska et al. 2017) demonstrated that the intracellular level of GSH and l-cysteine in U87MG cells was higher than in SH-SY5Y cells. It appears that under the same culture conditions, U87MG cells are less sensitive to oxidative stress than SH-SY5Y cells. Liu et al. (2015) reported that cancer cells were highly dependent on methionine and cystine for survival and proliferation. Their results showed that methionine and cystine double deprivation exerted a synergistic action on elevating ROS level, decreasing GSH level and inhibition of glioma (U87 and U251) cell proliferation. In the co-culture system, glial U87 cells protected neuronal SH-SY5Y cells from the indirect effect of radiation by reducing oxidative stress and apoptosis (Saeed et al. 2015).

Isothiocyantaes, such as benzyl isothiocyanate and sulforaphene, efficiently reduce the survival of prostate and breast cancer cells, what is associated with the induction of ROS, disruption of mitochondrial membrane potential and downregulation of Bcl-2 protein (Lin et al. 2017; Pawlik et al. 2017). We have shown that the inhibition of SH-SY5Y and U87MG cells proliferation by HBITC treatment (Fig. 1) is also associated with a decrease in mitochondrial membrane potential (Fig. 7) and with an increase of the inactive form of Bcl-2 (Fig. 5). It is possible that sulfhydryl groups of Bcl-2 can be modified via S-sulfuration or oxidative stress, as we also reported previously (Jurkowska et al. 2017). Interestingly, we have observed in SH-SY5Y cells downregulation of p53 and upregulation of p21 protein levels after HBITC treatment (Fig. 4). Both SH-SY5Y and U87MG cancer cell lines contain wild-type p53 protein (Mustafa Rizvi et al. 2014; Janardhanan et al. 2009; Cerrato et al. 2001; Giacoppo et al. 2017). It seems that inhibition of p53 protein in SH-SY5Y cells can be also associated with S-sulfuration or induction of oxidative stress in these cells. The cysteine residues located at the surface of p53 protein (Cys 124, Cys 176, Cys 182, Cys 229, Cys 242, and Cys 277) function as redox sensors (Kim et al. 2011; Bykov et al. 2016). It was reported that Cys 277 was essential for sequence specific DNA binding and a target for redox regulation of p53 (Buzek et al. 2002). p53 cysteine thiol modification is inversely correlated with its DNA binding properties and transcriptional activity (Kim et al. 2011; Liu et al. 2008). When p53-deficient human lung cancer NCI-H358 cells were transfected with wild-type p53 and subsequently challenged with H_2_O_2_, decreased DNA binding properties as well as reduced transcriptional activity of p53 were observed (Parks et al. 1997). Giacoppo et al. (2017) showed that treatment of SH-SY5Y cells with the moringa isothiocyanate complexed with α-cyclodextrin (MAC complex) caused inhibition of proliferation and induction of p21 protein, but p53 protein level was decreased at low concentrations of MAC complex. Moreover, Mustafa Rizvi et al. (2014) reported that aluminum-induced apoptosis of SH-SY5Y cells was correlated with increasing ROS production and intracellular calcium level, and with downregulation of p53 protein level.

Our data strongly suggest that upregulation of p21 protein level in SH-SY5Y cells treated with HBITC occurs in a p53-independent manner. In addition to the well-known p53-dependent p21 activation, p21 can be activated by a variety of molecules, i.e. specificity protein 1 (Sp1), histone deacetylase, activating protein-2 (AP-2), elongation 2 factors (E2Fs), signal transducers and activators of transcription (STATs) (Piccolo and Crispi 2012; Roninson 2002). It is known that isothiocyanates are the histone deacetylase inhibitors (Nian et al. 2009). It is, therefore, possible that HBITC treatment results in p21 protein expression level in SH-SY5Y cells, because it affects the histone deacetylase activity.

## Conclusions

**Fig. 8:**
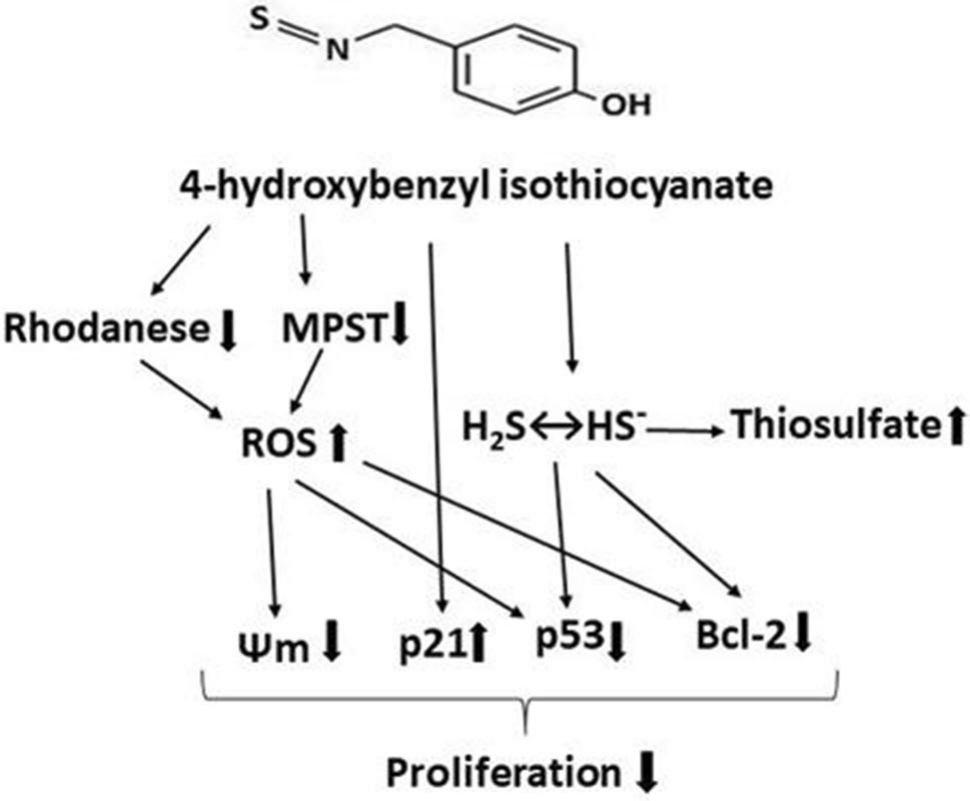
The antiproliferative activities of 4-hydroxybenzyl isothiocyanate

4-Hydroxybenzyl isothiocyanate, a natural H_2_S-donor, exerts the antiproliferative effect on SH-SY5Y and U87MG cells. Changes of mitochondrial TST and MPST levels, an increased ROS level, and changes of the level of cell cycle proteins in HBITC-treated SH-SY5Y cells, but not in U87MG cells, are associated with a lower mitochondrial metabolic capacity (Kim et al. 2015) and with a lower level of GSH (Jurkowska et al. 2017) in SH-SY5Y cells. Downregulation of mitochondrial TST and MPST protein levels in the presence of HBITC can result in an increased ROS level in SH-SY5Y cells (Fig. 8). The sulfhydryl groups of p53 protein could be modified via HBITC-induced oxidative stress in SH-SY5Y cells, what can trigger a decrease in p53 protein level. It is possible that inactivation of p53 protein in HBITC-treated SH-SY5Y cells can be also caused by its S-sulfuration in the presence of high levels of H_2_S and thiosulfate. Interestingly, it seems that the induction of p21 protein level in SH-SY5Y cells is associated with p53-independent mechanisms. In both (SH-SY5Y and U87MG) cancer cells, HBITC affects a decrease in mitochondrial membrane potential and number of cells with activated Bcl-2 protein (inactivation of Bcl-2 protein via HBITC induced S-sulfuration or oxidative stress) (Fig. 8).

## Abbreviations

SH-SY5Y: The human neuroblastoma cell line
U87MG: The human glioblastoma cell line
HBITC: 4-Hydroxybenzyl isothiocyanate
TST: Rhodanese
CTH: Cystathionine-γ-lyase
CBS: Cystathionine-β-synthase
MPST: 3-Mercaptopyruvate sulfurtransferase
Bcl-2: B-cell lymphoma-2
MMP: Mitochondrial membrane potential
H_2_S: Hydrogen sulfide
GS-SH: Glutathione persulfide
SQR: Sulfide:quinone oxidoreductase
GSH: Reduced glutathione
ROS: Reactive oxygen species
PPAR-γ: Peroxisome proliferator-activated receptor-γ
LDH: Lactate dehydrogenase
Sp1: Specificity protein 1
AP-2: Activating protein-2
E2Fs: Elongation 2 factor*s*
STATs: Signal transducers and activators of transcription

## References

[CR1] Baskar R, Bian J (2011). Hydrogen sulfide gas has cell growth regulatory role. Eur J Pharmacol.

[CR2] Buzek J, Latonen L, Kurki S, Peltonen K, Laiho M (2002). Redox state of tumor suppressor p53 regulates its sequencespecific DNA binding in DNA-damaged cells by cysteine 277. Nucleic Acids Res.

[CR3] Bykov VJ, Zhang Q, Zhang M, Ceder S, Abrahmsen L, Wiman KG (2016). Targeting of mutant p53 and the cellular redox balance by APR-246 as a strategy for efficient cancer therapy. Front Oncol.

[CR4] Cerrato JA, Yung WK, Liu TJ (2001). Introduction of mutant p53 into a wild-type p53 expressing glioma cell line confers sensitivity to Ad-p53-induced apoptosis. Neuro Oncol.

[CR5] Citi V, Martelli A, Testai L, Marino A, Breschi MC, Calderone V (2014). Hydrogen sulfide releasing capacity of natural isothiocyanates: is it a reliable explanation for the multiple biological effects of Brassicaceae?. Planta Med.

[CR6] Dufour V, Stahl M, Baysse C (2015). The antibacterial properties of isothiocyanates. Microbiology.

[CR7] Giacoppo S, Iori R, Rollin P, Bramanti P, Mazzon E (2017). Moringa isothiocyanate complexed with α-cyclodextrin: a new perspective in neuroblastoma treatment. BMC Complement Altern Med.

[CR8] Gillies RJ, Didier N, Denton M (1986). Determination of cell number in monolayer cultures. Anal Biochem.

[CR9] Gupta P, Kim B, Kim SH, Srivastava SK (2014). Molecular targets of isothiocyanates in cancer: recent advances. Mol Nutr Food Res.

[CR10] Gupta P, Wright SE, Kim SH, Srivastava SK (2014). Phenethyl isothiocyanate: a comprehensive review of anti-cancer mechanisms. Biochim Biophys Acta.

[CR11] Hellmich MR, Coletta C, Chao C, Szabo C (2015). The therapeutic potential of cystathionine β-synthetase/hydrogen sulfide inhibition in cancer. Antioxid Redox Signal.

[CR12] Jadhav U, Ezhilarasan R, Vaughn SF, Berhow MA, Mohanam S (2007). Iberin induces cell cycle arrest and apoptosis in human neuroblastoma cells. Int J Mol Med.

[CR13] Janardhanan R, Banik NL, Ray SK (2009). *N*-Myc downregulation induced differentiation, early cell cycle exit, and apoptosis in human malignant neuroblastoma cells having wild type or mutant p53. Biochem Pharmacol.

[CR14] Jurkowska H, Uchacz T, Roberts J, Wróbel M (2011). Potential therapeutic advantage of ribose-cysteine in the inhibition of astrocytoma cell proliferation. Amino Acids.

[CR15] Jurkowska H, Roman HB, Hirschberger LL, Sasakura K, Nagano T, Hanaoka K, Krijt J, Stipanuk MH (2014). Primary hepatocytes from mice lacking cysteine dioxygenase show increased cysteine concentrations and higher rates of metabolism of cysteine to hydrogen sulfide and thiosulfate. Amino Acids.

[CR16] Jurkowska H, Wróbel M, Kaczor-Kamińska M, Jasek-Gajda E (2017). A possible mechanism of inhibition of U87MG and SH-SY5Y cancer cell proliferation by diallyl trisulfide and other aspects of its activity. Amino Acids.

[CR17] Kabil O, Vitvitsky V, Banerjee R (2014). Sulfur as a signaling nutrient through hydrogen sulfide. Annu Rev Nutr.

[CR18] Kim DH, Kundu JK, Surh YJ (2011). Redox modulation of p53: mechanisms and functional significance. Mol Carcinog.

[CR19] Kim J, Han J, Jang Y, Kim SJ, Lee MJ, Ryu MJ, Kweon GR, Heo JY (2015). High-capacity glycolytic and mitochondrial oxidative metabolisms mediate the growth ability of glioblastoma. Int J Oncol.

[CR20] Lai KC, Huang AC, Hsu SC, Kuo CL, Yang JS, Wu SH, Chung JG (2010). Benzyl isothiocyanate (BITC) inhibits migration and invasion of human colon cancer HT29 cells by inhibiting matrix metalloproteinase-2/-9 and urokinase plasminogen (uPA) through PKC and MAPK signaling pathway. J Agric Food Chem.

[CR21] Lee ZW, Teo XY, Tay EY, Tan CH, Hagen T, Moore PK, Deng LW (2014). Utilizing hydrogen sulfide as a novel anti-cancer agent by targeting cancer glycolysis and pH imbalance. Br J Pharmacol.

[CR22] Lin JF, Tsai TF, Yang SC, Lin YC, Chen HE, Chou KY, Hwang TI (2017). Benzyl isothiocyanate induces reactive oxygen species-initiated autophagy and apoptosis in human prostate cancer cells. Oncotarget.

[CR23] Liu B, Chen Y, St Clair DK (2008). ROS and p53: a versatile partnership. Free Radic Biol Med.

[CR24] Liu H, Zhang W, Wang K, Wang X, Yin F, Li C, Wang C, Zhao B, Zhong C, Zhang J, Peng F, Bi Y, Shen C, Hou X, Zhang D, Liu Y, Ai J, Zhao S (2015). Methionine and cysteine double deprivation stress suppresses glioma proliferation via inducing ROS/autophagy. Toxicol Lett.

[CR25] Marutani E, Yamada M, Ida T, Tokuda K, Ikeda K, Kai S, Shirozu K, Hayashida K, Kosugi S, Hanaoka K, Kaneki M, Akaike T, Ichinose F (2015). Thiosulfate mediates cytoprotective effects of hydrogen sulfide against neuronal ischemia. J Am Heart Assoc.

[CR26] Meng G, Zhao S, Xie L, Han Y, Ji Y (2017). Protein S-sulfhydration by hydrogen sulfide in cardiovascular system. Br J Pharmacol.

[CR27] Mustafa Rizvi SH, Parveen A, Verma AK, Ahmad I, Arshad M, Mahdi AA (2014). Aluminium induced endoplasmic reticulum stress mediated cell death in SH-SY5Y neuroblastoma cell line is independent of p53. PLoS ONE.

[CR28] Mustafa AK, Gadalla MM, Sen N, Kim S, Mu W, Gazi SK, Barrow RK, Yang G, Wang R, Snyder SH (2009). H_2_S signals through protein S-sulfhydration. Sci Signal.

[CR29] Nagahara N (2011). Catalytic site cysteines of thiol enzyme: sulfurtransferases. J Amino Acids.

[CR30] Nagahara N, Nagano M, Ito T, Suzuki H (2015). Redox regulation of mammalian 3-mercaptopyruvate sulfurtransferase. Methods Enzymol.

[CR31] Nian H, Delage B, Ho E, Dashwood RH (2009). Modulation of histone deacetylase activity by dietary isothiocyanates and allyl sulfides: studies with sulforaphane and garlic organosulfur compounds. Environ Mol Mutagen.

[CR32] Parks D, Bolinger R, Mann K (1997). Redox state regulates binding of p53 to sequence-specific DNA, but not to non-specific or mismatched DNA. Nucleic Acids Res.

[CR33] Pawlik A, Wała M, Hać A, Felczykowska A, Herman-Antosiewicz A (2017). Sulforaphene, an isothiocyanate present in radish plants, inhibits proliferation of human breast cancer cells. Phytomedicine.

[CR34] Peng B, Chen W, Liu C, Rosser EW, Pacheco A, Zhao Y, Aguilar HC, Xian M (2014). Fluorescent probes based on nucleophilic substitution-cyclization for hydrogen sulfide detection and bioimaging. Chem Eur J.

[CR35] Piccolo MT, Crispi S (2012). The dual role played by p21 may influence the apoptotic or anti-apoptotic fate in cancer. J Can Res Updates.

[CR36] Roninson IB (2002). Oncogenic functions of tumour suppressor p21Waf1/Cip1/Sdi1: association with cell senescence and tumour-promoting activities of stromal fibroblasts. Cancer Lett.

[CR37] Rose P, Moore PK, Zhu YZ (2017). H_2_S biosynthesis and catabolism: new insights from molecular studies. Cell Mol Life Sci.

[CR38] Saeed Y, Xie B, Xu J, Rehman A, Hong M, Hong Q, Deng Y (2015). Glial U87 cells protect neuronal SH-SY5Y cells from indirect effect of radiation by reducing oxidative stress and apoptosis. Acta Biochim Biophys Sin.

[CR39] Shih VE, Carney MM, Mandell R (1979). A simple screening test for sulfite oxidase deficiency: detection of urinary thiosulfate by a modification of Sorbo’s method. Clin Chim Acta.

[CR40] Song ZJ, Ng MY, Lee ZW, Dai W, Hagen T, Moore PK, Huang D, Deng LW, Tan CH (2014). Hydrogen sulfide donors in research and drug development. Med Chem Commun.

[CR41] Su JC, Lin K, Wang Y, Sui SH, Gao ZY, Wang ZG (2015). In vitro studies of phenethyl isothiocyanate against the growth of LN229 human glioma cells. Int J Clin Exp Pathol.

[CR42] Szabo C, Papapetropoulos A (2017). International union of basic and clinical pharmacology. CII: pharmacological modulation of H_2_S levels: H_2_S donors and H_2_S biosynthesis inhibitors. Pharmacol Rev.

[CR43] Tkacheva NI, Morozov SV, Lomivorotov BB, Grigor’ev IA (2017). Molecular biological problems of drug design and mechanism of drug action. Organic hydrogen sulfide donor compounds with cardioprotective properties (Review). Pharm Chem J.

[CR44] Toohey JI (2012). The conversion of H_2_S to sulfane sulfur. Nature Rev Mol Cell Biol.

[CR45] Yagdi E, Cerella C, Dicato M, Diederich M (2016). Garlic-derived natural polysulfanes as hydrogen sulfide donors: friend or foe?. Food Chem Toxicol.

[CR46] Zhang JY, Ding YP, Wang Z, Kong Y, Gao R, Chen G (2017). Hydrogen sulfide therapy in brain diseases: from bench to bedside. Med Gas Res.

[CR47] Zhang D, Du J, Tang C, Huang Y, Jin H (2017). H_2_S-induced sulfhydration: biological function and detection methodology. Front Pharmacol.

[CR48] Zheng Y, Ji X, Ji K, Wang B (2015). Hydrogen sulfide prodrugs—a review. Acta Pharm Sin B.

[CR49] Zhu Y, Zhuang JX, Wang Q, Zhang HY, Yang P (2013). Inhibitory effect of benzyl isothiocyanate on proliferation in vitro of human glioma cells. Asian Pac J Cancer Prev.

